# Oak Acorns as Functional Foods: Antioxidant Potential and Safety Assessment

**DOI:** 10.3390/foods14142486

**Published:** 2025-07-16

**Authors:** Vesna Stankov Jovanović, Vladan Djurić, Violeta Mitić, Ana Barjaktarević, Snežana Cupara, Marija Ilić, Jelena Nikolić

**Affiliations:** 1 Department of Chemistry, Faculty of Science and Mathematics, University of Niš, Višegradska 33, 18000 Niš, Serbia; violeta.mitic@pmf.edu.rs (V.M.); jelena.cvetkovic@pmf.edu.rs (J.N.); 2 Faculty of Agriculture, University of Priština in Kosovska Mitrovica, Kopaonička bb, 38228 Lešak, Serbia; 3 Department of Pharmacy, Faculty of Medical Sciences, University of Kragujevac, Svetozara Markovića 69, 34000 Kragujevac, Serbia; ana.radovanovickg@gmail.com (A.B.); snezanacupara@yahoo.com (S.C.); 4 Specialized Veterinary Institute, Dimitrija Tucovića 175, 18000 Niš, Serbia; marija.fertico@gmail.com

**Keywords:** coffee substitute, oak acorn flour, oak acorn coffee, antioxidant activity, PAH content, food safety

## Abstract

With the growing interest in natural and health-supporting foods, oak acorns (*Quercus robur*) are gaining renewed attention for their nutritional and antioxidant potential. This study explored how different processing methods affect bioactive compounds in three acorn-based products: raw acorn flour, roasted “coffee,” and washed-and-roasted “super coffee.” Extracts were obtained using methanol, acetone, and hexane to evaluate total phenolic content (TPC), total flavonoid content (TFC), and antioxidant activity via ABTS, DPPH, CUPRAC, FRAP, and TRP assays. Methanol proved to be the most effective solvent, extracting up to 66.53 mg GAE/g dw of phenolics in raw flour and 76.50 mg GAE/g dw in roasted “coffee,” reflecting a 15% increase in TPC after thermal treatment. However, the same treatment resulted in a 17% decrease in flavonoid content, from 181.5 mg RE/g dw in raw flour to 150.67 mg RE/g dw in “super coffee.” Antioxidant activity followed a similar pattern, with methanol extracts showing the highest values, up to 584 mg TE/g dw in the CUPRAC assay and 126.7 mg TE/g dw in ABTS. Safety was also assessed through the quantification of 16 priority polycyclic aromatic hydrocarbons (PAHs). The total PAH levels in the roasted “coffee” and “super coffee” samples were 222 ng/g dw and 290 ng/g dw, respectively. Importantly, PAH4 compounds, used as key safety indicators in EU regulations, were present in low concentrations, primarily as benzo[a]anthracene (34.3–39.8 ng/g), and none exceeded the maximum limits established for cocoa-based products. Benzo[a]pyrene, a major carcinogen, was not detected. The results confirm that acorns of *Quercus robur*, especially in their native flour form, are rich in antioxidants, naturally gluten-free, and safe when thermally processed, making them a strong candidate for use in functional foods.

## 1. Introduction

Acorns, the nuts of oak trees (*Quercus* spp.), have been valued for centuries across many cultures as both a staple food and a traditional remedy. Members of the Fagaceae family, *Quercus* species, are widely distributed across Europe, North America, and Asia. While historically, acorns formed a significant part of the human diet, their consumption has declined, largely due to their high tannin content, which imparts bitterness and impairs nutrient absorption by binding to proteins and minerals [1,2].

However, with the rising global interest in sustainable, health-promoting foods, acorns have reemerged as candidates for functional food development. Acorn flour, in particular, is naturally gluten-free and rich in fiber, and it may enhance the nutritional and sensory properties of baked goods. Several studies have demonstrated the successful incorporation of acorn flour into various products, including bread, cakes, and cookies [3,4].

Coffee remains one of the most widely consumed beverages worldwide, appreciated for its aroma, flavor, and stimulating effects. Its antioxidant activity is well established and attributed to its content of polyphenols, alkaloids (caffeine), and diterpenes [5,6,7].

In recent years, driven by health consciousness and dietary restrictions, coffee substitutes have gained popularity. These include chicory, barley, spelt, dandelion, and notably, acorns [8]. Such alternatives provide fiber, vitamins, minerals, and phenolic acids like gallic, caffeic, and chlorogenic acid [9,10,11,12,13].

Historically, acorns were commonly used as substitutes for coffee during periods of scarcity, such as wartime. Today, their appeal lies in their affordability, sustainability, and promising nutritional profile. Species from the Quercus genus have shown a wide range of bioactivities, including antioxidant, antimicrobial, and anticancer properties [2,14]. Phytochemical analyses have revealed that acorns are rich in phenolic acids (e.g., gallic, chlorogenic, ellagic), flavonoids (e.g., catechin, quercetin), and tannins (e.g., vescalagin, castalagin, roburin) [15,16,17].

Beyond polyphenols, acorns contain essential nutrients—carbohydrates, dietary fiber, lipids, and proteins—making them an intriguing ingredient for both food and health sectors. Their lipid fraction is especially rich in unsaturated fatty acids, such as oleic and linoleic acids, while small amounts of vitamin E and A precursors have also been identified [18,19,20]. Acorn flours have been reported to contain 41–79% carbohydrates (mostly starch), moderate protein (2–5%), and substantial fiber (13–52% dw) [21,22]. The oil content in *Q. robur* has been measured at 52–56 mg/g dry weight (dw), with approximately 79% as unsaturated fatty acids [23].

Perhaps most importantly, acorns are gluten-free and contain prebiotic polysaccharides, offering a valuable alternative to cereal for individuals with gluten sensitivities and celiac disease [24,25,26]. This feature alone supports their inclusion in specialized diets and functional foods.

Despite these promising attributes, relatively few studies have examined the antioxidant activity of *Q. robur* fruits, particularly in coffee-like beverages made from roasted acorns. Available studies tend to focus on specific solvent extracts (e.g., methanol, ethanol, or water) rather than on products resembling coffee infusions [27,28]. For instance, studies have shown that extracts from *Q. robur* leaves, seeds, and shells possess high antioxidant potential, particularly when evaluated using DPPH, ABTS, or FRAP assays [29,30].

Acorn roasting, a common pre-treatment to reduce bitterness and enhance flavor, alters the chemical profile of the seeds. Tannin levels decline significantly, while the degradation of complex phenolics may increase the content of simpler antioxidant compounds such as gallic acid [31,32]. Samsonowicz et al. demonstrated that acorn-based beverages, especially those combined with other herbs such as ginseng, contain a high total polyphenol content and exhibit strong antioxidant potential compared to commercial coffee alternatives [33].

However, thermal processing also carries potential health risks. Roasting at high temperatures (typically above 200 °C) can induce the formation of polycyclic aromatic hydrocarbons (PAHs), a class of lipophilic environmental pollutants formed through the pyrolysis of organic matter. PAHs are known for their mutagenic and carcinogenic properties [34,35]. PAHs can also be introduced during food processing methods like smoking, frying, or drying [36]. The most toxic PAHs—those with five or more aromatic rings—are classified as high-molecular-weight PAHs (HMW-PAHs) and include compounds such as benzo[a]pyrene and benzo[b]fluoranthene [37,38].

The U.S. Environmental Protection Agency (EPA) has identified 16 PAHs as priority pollutants, while the European Commission has issued regulations specifying the maximum allowable limits for four of the most toxic PAHs (PAH4: benzo[a]pyrene, chrysene, benzo[a]anthracene, benzo[b]fluoranthene) in cocoa products but not in coffee [39,40,41].

Although coffee and coffee substitutes are exempt from EU PAH limits, studies have shown that roasting acorns can result in PAH concentrations, especially of anthracene and benzo[a]anthracene, which merit attention [42]. Notably, acorn-based beverages may contain lower PAH levels than conventional coffee, potentially offering a safer alternative, especially if the roasting process is carefully optimized [43].

Given this duality—high antioxidant benefits versus potential PAH contamination—a comprehensive evaluation is essential for understanding the benefit-to-risk profile of roasted acorns used as coffee substitutes.

This study uniquely examines the antioxidant potential and PAH content of coffee-like beverages prepared from roasted *Q. robur* acorns. Previous research either focused on phenolic composition or antioxidant activity alone. Here, we simultaneously assess the total phenolics, flavonoids, and antioxidant capacity using five complementary assays (ABTS, DPPH, CUPRAC, FRAP, and TRP), along with the quantification of all 16 priority PAHs and an evaluation of safety regarding the 4 PAHs that are carcinogenic and potentially carcinogenic.

This integrated approach enables a more holistic evaluation of roasted acorn products, comparing their nutritional benefits against the safety risks associated with PAH exposure. Moreover, by focusing on a botanically defined species, *Q. robur*, this study provides reproducible, species-specific insights critical for food safety, dietary recommendations, and future product development.

## 2. Materials and Methods

### 2.1. Chemicals and Instruments

For the quantitative analysis of 16 priority PAHs, a PAH Kit 601–N (Supelco, Bellefonte, PA, USA) containing 16 EPA PAHs was used as the standard sample. The internal standards (acenaphthene d_10_ and chrysene d_10_) used for the quantification of 16 priority PAHs, i.e., p-terphenyl d_14_ used as a surrogate standard, were purchased from Supelco, Bellefonte, PA, USA.

All samples for PAH content were analyzed on a 7890/7000B GC–MS/MS system (Agilent Technologies, Santa Clara, CA, USA, equipped with a Combi PAL autosampler) in the selected ion monitoring (SIM) mode based on the use of one ion.

This research utilized extracts from three solvents of varying polarities—methanol, acetone, and hexane—to test hydrophilic antioxidants that are soluble in polar solvents and lipophilic antioxidants. The relative polarity of methanol, acetone, and hexane is 0.762, 0.355, and 0.009, respectively. Solvents were purchased from Sigma Aldrich, Taufkirchen, Germany.

2,2-Diphenyl-1-picrylhydrazyl (DPPH), 2,2′-Azino-bis(3-ethylbenzothiazoline-6-sulphonic acid) (ABTS), iron(III) chloride hexahydrate, Folin–Ciocalteu reagent, gallic acid (3,4,5-trihydroxybenzoic acid), 6-hydroxy-2,5,7,8-tetramethylchroman-2-carboxylic acid (Trolox), ascorbic acid, and methanol were purchased from Sigma Co., St. Louis, MO, USA.

The following chemical substances of analytical grade were supplied from the corresponding sources: neocuproine (2,9-dimethyl-1,10-phenanthroline), copper(II) chloride dihydrate, NaCO_3_, HCl, 2,4,2-tri(2-pyridyl)-s-triazine (TPTZ), K_3_[Fe(CN)_6_], phosphate buffer (NaH_2_PO_4_–Na_2_HPO_4_), ammonium acetate buffer, CCl_3_COOH, K_2_S_2_O_8_, FeSO_4_·7H_2_O, and DMSO (dimethyl sulphoxide), which were purchased from Merck, Darmstadt, Germany.

Spectrophotometric assays were performed using a double-beam UV–Vis spectrophotometer, Lambda 15, PerkinElmer, Waltham, MA, USA. Each of the oak samples was analyzed in triplicate.

### 2.2. Sample Preparation

Oak (*Q. robur*) acorns were collected under individual oak trees in October and November 2022 at the Kosmaj mountain (central Serbia, Latitude 44°33′56″; Longitude 44°27′56″). Generally, 90–100 acorns were taken, air-dried at room temperature (23–25 °C), and shelled out. About 100 g of the dry-shelled acorns were powdered using a commercial grinder mill brand, “Bosch”, Gerlingen, Baden-Württemberg, Germany. The flour obtained in this way was used for phytochemical analysis.

A mass of 100 g of dry acorns was exposed to heat (200 °C) for 30 min and left to cool until being milled to powder (1–2 mm)—“coffee”.

The dry-shelled acorns (100 g) were coarsely chopped into 0.2–0.5 cm pieces and soaked in heated water (60–70 °C) for 30 min. The water was discarded, and the soaking process was repeated, adding a new quantity of heated water. The washing procedure was repeated four times. The resulting chopped and soaked pieces of oak acorns were then dried at 40 °C for 2–5 h, depending on the material in the batch and the thickness of the layer, until a constant mass was achieved. Afterward, the material was exposed to the heat (200 °C) for 30 min, cooled, and milled to powder (1–2 mm)—“super coffee” [44].

### 2.3. Preparation of Extracts for Antioxidant Analysis

A total of 10 g of powdered “acorn coffee” and flour were extracted two times by stirring with 100 mL of three solvents of various polarity (methanol, acetone, hexane) in an ultrasonic bath for 15 min at 25 °C. Samples were left in the solvent overnight, filtered, and evaporated to dryness using a vacuum rotary evaporator. The exact masses of the dry extracts were dissolved in dimethyl sulphoxide (DMSO) to a final concentration of 50 mg/mL [45].

### 2.4. Total Phenolic Content (TPC)

Phenolic compounds mainly represent the secondary metabolites of more complex compounds. The total reducing phenolic content was tested using the Folin–Ciocalteu reagent [45].

The reaction mixture was prepared using 0.05 mL of extract, 2 mL of 20% sodium carbonate solution, 0.5 mL of the Folin–Ciocalteu reagent, and 5.0 mL of distilled water to a final volume of 7.55 mL. The reaction was carried out in the dark for 30 min, and then the absorbance was measured at 750 nm. The results were expressed as mg of gallic acid equivalents per 1 g of dry extract weight (mg GAE/g dw) since gallic acid was used to calculate the standard curve (y = 0.0163 x + 0.0121, R^2^ = 0.98).

### 2.5. Total Flavonoid Content (TFC)

The total flavonoid content of prepared extracts was determined by a method described by Mitic et al. [46].

An aliquot of extract (0.05 mL) was mixed with 0.15 mL of a 5% NaNO_2_ solution, followed by the addition of 0.75 mL of 2% AlCl_3_ solution. The prepared solution was kept at room temperature for 5 min, and then 1 mL of 1 mol/L NaOH solution was added to the mixture. Water was added to bring the final volume to 5 mL. The absorbance was measured at 510 nm. Rutin solution was used for calibration curve construction (y = 0.0356x + 0.0214 R^2^ = 0.99), and results were expressed as mg rutin equivalents (RE) per 1 g of dry extract weight (mg RE/g dw).

### 2.6. Antioxidant Activity

Due to the chemical diversity of antioxidant molecules and their mechanisms of action, several methods have been developed to determine the antioxidant capacity of various samples. Researchers often use several techniques based on different reaction mechanisms [47].

### 2.7. DPPH “Scavenging” Radical Capacity

The extracts’ quantitative assays on DPPH radicals were performed according to the method of Nikolic et al. [45].

A volume of 1.5 mL of DPPH radical methanol solution (100 mmol/L), 0.05 mL of extract, and methanol to a total volume of 4 mL were added to a test tube. The mixture was shaken and left in the dark at room temperature for 60 min. The absorbance was measured at 515 nm wavelength, and results were expressed as mg of trolox equivalents (TE).

Trolox solution was used for calibration curve construction (y = 0.0405x − 0.0495, R^2^ = 0.99), and results were expressed as mg of trolox equivalents (TE) per 1 g of dry extract weight (mg TE/g dw).

### 2.8. ABTS Radical “Scavenging” Activity

ABTS radical “scavenging” activity was performed according to the method of Nikolic et al. [45]. A solution of ABTS in methanol with a concentration of 7 mmol/L was prepared. An ABTS^∙+^ radical cation is formed as a product of the reaction between ABTS solution and 2.4 mmol/L K_2_S_2_O_8_ solution. A working solution is prepared by mixing these two in a 1:1 ratio (10 mL of each). It is left to stand in the dark at room temperature for 12 h before use. A volume of 14.8 mL of this solution was diluted with 240 mL of methanol to achieve an absorbance of the resulting solution of 0.700 ± 0.02 at a wavelength of 734 nm. A volume of 1.8 mL of ABTS radical methanol solution, 0.05 mL of extract, and methanol to a total volume of 4 mL were added to a test tube. The mixture was shaken and left in the dark at room temperature for 6 min. The reduction in absorbance was measured at a wavelength of 734 nm. Results were expressed as mg of Trolox equivalents (TE).

Trolox solution was used for calibration curve construction (y = 0.0322x − 0.0141, R^2^ = 0.99), and results were expressed as mg of trolox equivalents (TE) per 1 g of dry extract weight (mg TE/g dw).

### 2.9. Cupric Reducing Antioxidant Capacity (CUPRAC) Assay

The CUPRAC assay was performed using the method of Nikolic et al. [45].

Volume of 0.05 mL of the extract, 1 mL of phosphate buffer (pH 7.0), 7.5 mmol/L of neocuproine, and 0.01 mol/L of copper (II) chloride were mixed, and the mixture was then diluted with water to a total volume of 4.1 mL. Reaction mixtures were incubated at room temperature for 30 min, and absorbance was measured at 450 nm. Trolox solution was used for calibration curve construction (y = 0.0959x − 0.0249, R^2^ = 0.99), and results were expressed as mg of trolox equivalents (TE) per 1 g of dry extract weight (mg TE/g dw).

### 2.10. Ferric-Reducing Antioxidant Power (FRAP) Assay

A FRAP assay was performed using the method developed by Nikolic et al. [45].

FRAP reagent was prepared by mixing 200 mL of acetate buffer (pH 3.6) with 20 mL of 2,4,6-tris(2-pyridyl)-s-triazine solution (0.25 mmol of TPTZ dissolved in 10 mL of 40 mM HCl) and 20 mL of FeCl_3_. An aliquot of the extract (50 μL) was mixed with 3 mL of the FRAP solution and diluted with water to a final volume of 4 mL. After incubation of the mixture at 37 °C (for 5 min), the absorbance at 595 nm wavelength was recorded. The FRAP values were determined by plotting on a standard curve (y = 0.0955x − 0.0185, R^2^ = 0.9941), and results were expressed as mg of Fe equivalents (Fe) per 1 g of dry extract weight (mg Fe/g dw).

### 2.11. Total Reducing Power (TRP) Assay

The reducing power of extracts was determined by Nikolic et al.’s method [45].

Reaction mixtures were prepared by mixing 1 mL of a 1% solution of K_3_[Fe(CN)_6_], 1 mL of phosphate buffer (pH 6.6), and 0.01 mL of the extract and diluting with water to 3.7 mL. The mixtures were incubated at 50 °C for 30 min, and then 1 mL of a 10% solution of trichloroacetic acid and 0.6 mL of 0.1% FeCl_3_ were added. The absorbance was measured at a wavelength of 700 nm against the blank sample.

Ascorbic acid solution was used for calibration curve construction (y = 0.1056x − 0.0452, R^2^ = 0.98), and results were expressed as mg of ascorbic acid equivalents (AAE) per 1 g of dry extract weight (mg AAE/g dw).

### 2.12. PAH Analysis

#### 2.12.1. Sample Preparation for PAH Content Determination

Oak samples were air-dried in the dark room to prevent additional contamination. They were then stored in sealed bags at 4 °C until analysis.

The extraction of PAHs from samples was performed using the QuEChERS (Quick, Easy, Cheap, Effective, Rugged, and Safe) methodology [48]. All homogenized samples (10 g each) were mixed with water (15 mL) in QuEChERS tubes. After that, 15 mL of CH_3_CN and 0.1 mL of a surrogate standard containing p-terphenyl-d_14_, at a concentration of 100 μg/mL, were added to each extraction tube, followed by a minute of shaking. During the next step, 8 g of MgSO_4_ and 2 g of NaCl were added. The mixtures were shaken vigorously for 1 min, and the extracts were centrifuged at 3500 rpm for 10 min, allowing removal of the upper layers. Portions of 1 mL of the CH_3_CN layers were transferred into clean-up tubes containing 150 mg of MgSO_4_ and 50 mg of PSA. After shaking for 5 min, the tubes were centrifuged at 8000 rpm for 10 min. Portions of the upper layer (0.6 mL) from each tube were placed in vials for further gas chromatography–mass spectrometry (GC–MS) analysis. Additionally, 0.2 mL of an internal standard solution containing chrysene-d_10_ and acenaphthene-d_10_, each at a concentration of 80 μg/mL, was added.

Prior to analyzing real samples, method validation was performed. Calibration curves of 16 PAHs were constructed using a series of PAH standard solutions with a concentration range of 0.017–16.667 µg/mL. In method optimization experiments, raw coffee samples, with a verified absence of PAHs, have been used for blank and spiked samples. GC–MS analysis of blank samples showed no potential interferences. The LOD was calculated as three times higher than the signal-to-noise ratio, while the LOQ was equal to 10 times the signal-to-noise level. LOD were in the range of 0.12–8.88 ng/g. Accuracy, expressed as recovery values, was in the range from 71% for acenaphthene to 121% for benzo[a]pyrene [49].

#### 2.12.2. GC–MS Analysis of PAHs

The analyzed compounds were identified based on their qualifier ions and retention times. Chromatographic separations were conducted using a HP-5MS (5% Phenyl Methyl Siloxane) column (30 m × 250 μm × 0.25 μm). The GC oven was operated with the following temperature program: 75 °C for 3 min, then ramped at 6 °C/min to 300 °C and held for 10 min. The total runtime was 50 min and 30 s. A volume of 2.5 μL of acetonitrile extract was injected in splitless mode. The carrier gas was helium with a flow of 1.0 mL/min. Post-run: back flash for 1.89 min at 300 °C with helium at 50 Psi using a medical gas marker. MS conditions were as follows: ionization voltage of 70 eV, acquisition mass range of 40–560, and scan time of 0.32 s. Quantitative analysis used quantifier ions corresponding to each PAH and previously recorded retention time [41].

All experiments were repeated five times, and the precision was evaluated regarding repeatability, expressed as relative standard deviation (RSD). PAH concentrations were determined using Mass Hunter QQQ Quantitative Analysis software (Version 12.1, Agilent Technologies, Santa Clara, CA, USA) and presented as the average of five replicates ± SD.

### 2.13. Statistical Analysis

Statistical analysis was performed using Statistica 8.0 software (StatSoft, Tulsa, OK, USA). A probability level of *p* < 0.05 was considered statistically significant. A correlation between antioxidant activity assays, total phenolic, and total flavonoid content was established using regression analysis at a 95% significance level (*p* < 0.05). The paired Student *t*-test was used to compare the characteristics of different samples at a probability level of *p* < 0.05, as statistically significant.

## 3. Results and Discussion

Due to their multiple hydroxyl groups, phenols and flavonoids in *Quercus robur* acorns are readily extracted by highly polar solvents such as methanol and aqueous acetone. These include gallic and ellagic acids, their galloyl glycosides, catechin and condensed tannins (procyanidins), and flavonoid glycosides such as quercetin-3-O-glucoside, rutin, and kaempferol derivatives [6].

**Figure 1:**
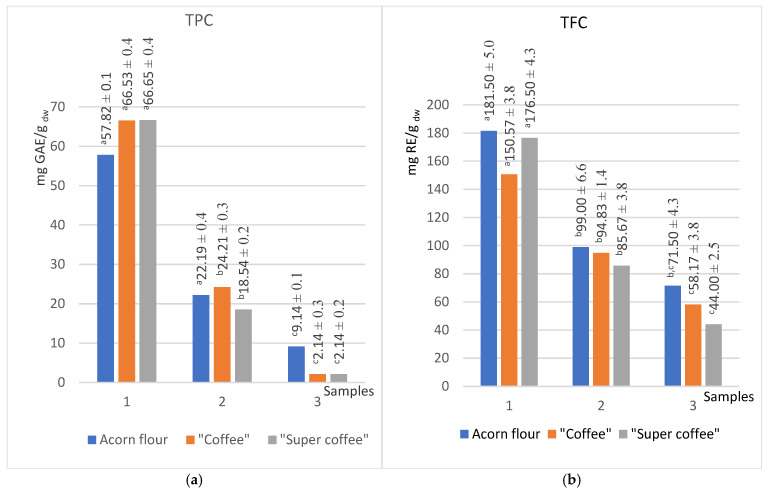
Content of total phenols (**a**) and flavonoids (**b**) in extracts of different polarities (methanol (1), acetone (2), hexane (3)) in the acorn flour, “coffee”, and “super coffee”. (Results marked with different letters (a, b, c) are significantly different at *p* < 0.05).

Since the polar nature of phenols and flavonoids contributed to the high value for the total content of these compounds, unsurprisingly, the highest values of total phenols and flavonoids were obtained in the series of methanol extracts. Acetone, as a less polar solvent, extracted a smaller amount of phenols and flavonoids; thus, the values for acetone extracts are lower than half of those for methanol. Hexane extracts, due to their low polarity, extracted minimal phenolics. For TPC, methanolic extracts reached 66.53 mg GAE/g dry weight (dw), while acetone extracts yielded 24.21 mg GAE/g dw, which is less than half (Figure 1a). An earlier study reported that TPC in aqueous coffee acorn extracts ranged from 45 to 50 mg GA/g dw [15]. Obtained values appear to align with previous studies reporting 45–50 mg GAE/g dry weight (dw) in aqueous acorn coffee extracts [26].

The total phenolic content (TPC) of aqueous acorn coffee extracts (45–50 mg GAE/g dw) falls between methanolic (66.5 mg GAE/g dw) and acetone extracts (24.2 mg GAE/g dw), consistent with solvent polarity effects reported in *Quercus* studies [50,51].

The highest TFC was also observed in the methanolic extracts of the acorn flour sample (181.50 mg RE/g dw), followed by “coffee” (161.72 mg RE/g dw) and “super coffee” (150.67 mg RE/g dw) (Figure 1b). Regardless of solvent polarity, the raw acorn flour consistently exhibited the highest flavonoid levels compared to its roasted counterparts, likely due to the partial degradation of thermolabile flavonoids during the roasting process.

The affirmative argument for using acorn “coffee” as a healthy substitute for natural coffee is that a similar total phenol content was found in commercially available coffee (37.26 GAE/g dw) and roasted coffee powder (42.6 mg GAE/g dw) [52,53].

Both thermally processed samples (“coffee” and “super coffee”) demonstrated a 15% and 9% increase in TPC (methanol and acetone extracts, respectively) relative to raw flour, suggesting the formation of low-molecular-weight phenolics during roasting. In contrast, TFC decreased by 17% in methanol and 4.2% in acetone extracts post-roasting, confirming the susceptibility of flavonoids to thermal degradation, which is in accordance with the result of Yazici et al. [20]

**Figure 2:**
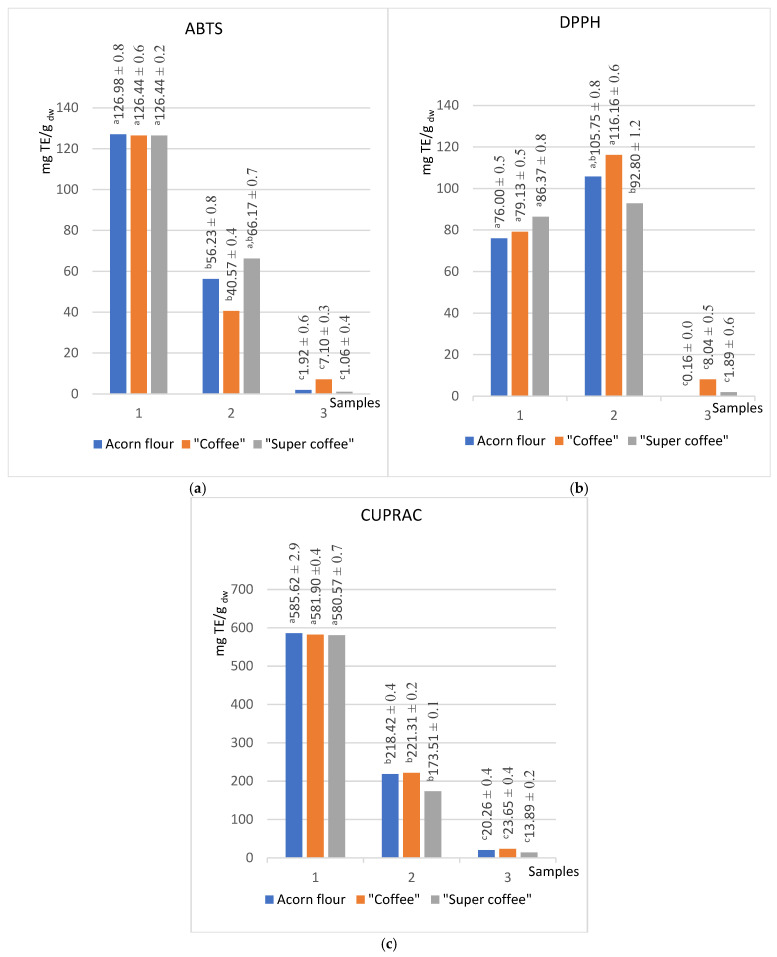
Radical scavenging activity of acorn products in extracts of different polarity (methanol (1), acetone (2), hexane (3)) obtained by ABTS (**a**), DPPH (**b**), and CUPRAC (**c**) methods. (Results marked with different letters (a, b, c) are significantly different at *p* < 0.05).

**Figure 3:**
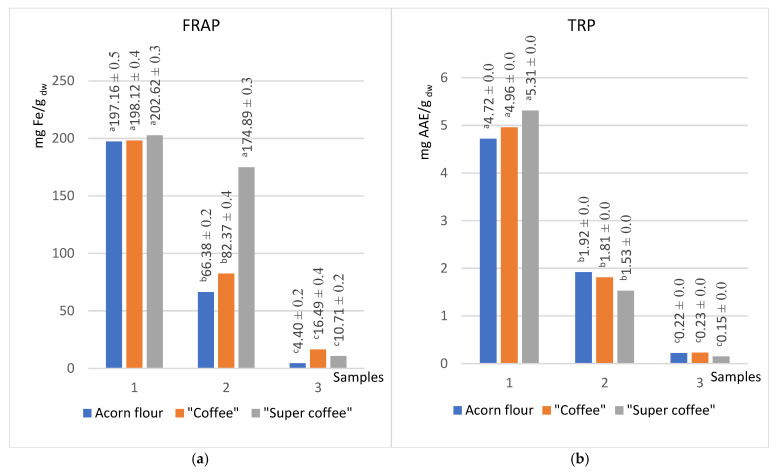
Reducing powers of acorn product extracts in solvents of different polarity (methanol (1), acetone (2), hexane (3)) obtained by FRAP (**a**) and TRP (**b**) methods (groups marked with different letters (a, b, c) are significantly different at *p* < 0.05).

This study employed a comprehensive approach to evaluate the antioxidant activity of acorn extracts, by applying ABTS, DPPH, and CUPRAC radical scavenging assays (Figure 2) and FRAP and TRP electron transfer assays (Figure 3) to ensure the robustness of the results.

Methanolic extracts consistently showed the highest antioxidant capacity across all methods, correlating with their elevated TPC and TFC levels (Figure 1). For instance, methanol extracts of all samples exhibited ABTS values of approximately 126.7 mg TE/g dw and CUPRAC values of 584 mg TE/g dw. In contrast, hexane extracts had the lowest antioxidant activity, reflecting minimal extraction of hydrophilic phenolics.

Notably, acetone extracts displayed slightly higher DPPH scavenging activity (104 mg TE/g dw) than methanol (81 mg TE/g dw), suggesting that certain mid-polarity antioxidants are better extracted by acetone. This divergence may reflect differences in radical selectivity between ABTS and DPPH assays or indicate solvent-specific phenolic profiles [5].

The CUPRAC method showed the highest sensitivity and strongest correlations with both TPC and TFC (r = 0.99 and 0.98, respectively). This method is advantageous for its broad applicability and has not been previously applied to acorn matrices, indicating a promising future research direction [17].

The FRAP assay demonstrated that thermal processing increased antioxidant power most dramatically in nonpolar extracts, with increases of +100.5%, +124.1%, and +374.8% in methanol, acetone, and hexane, respectively. This trend suggests the generation of thermally induced antioxidant compounds, such as Maillard reaction products or breakdown products of complex polyphenols, in roasted samples [20].

The antioxidant values of methanolic extracts obtained by the ABTS method are uniform for all samples analyzed. They amount to 126 ± 1 mg TE/g dw (Figure 2a), which is 12.7% higher than the value reported by Chavez et al. for regular coffee in the previous research—110 mg TE/g for the roasted coffee powder [52].

The copper (II)-neocuproine reagent of the CUPRAC test, known for its rapid reaction with a wide range of electron donors, was unaffected by the nature of antioxidants in acorns. This fact makes it less selective than the ABTS and DPPH tests, leading to significantly higher values of Trolox equivalents. Moreover, this method’s stability ensures consistent results across all three series. Notably, the values for “super coffee” decreased with increasing lipophilicity of the solvent compared to the other two samples. While the deviation of the obtained value for methanol is only −0.55% compared to the average value for flour and “coffee”, the values for acetone and hexane decrease by −21.09% and −36.75%, respectively (Figure 2c). It is worth mentioning that the CUPRAC method has yet to be applied to acorns, presenting an exciting avenue for future research.

The simple, rapid, cost-effective assay for determining ferric-reducing antioxidant power (FRAP) is a typical ET-based method performed under acidic pH conditions. The reducing capacity of antioxidants, measured by the FRAP method, increases in value after heat treatment, and these differences are more pronounced with a decrease in the polarity of the extracts (100.5%, 124.1%, and 374.8%, respectively, for methanol, acetone, and hexane). The reducing power assay is a convenient and rapid screening method for measuring the antioxidant potential. The reducing power of extracts is related to their electron transfer ability and may serve as a significant indicator of potential antioxidant activity.

The hexane extracts, as expected, showed the lowest antioxidant activities considering the low total contents of phenols and flavonoids. However, the “acorn coffee” stood out in this series, consistently demonstrating the highest values of antioxidant activity (Figure 2 and Figure 3). Interestingly, the total content of phenols and flavonoids was the highest in the raw substance (Figure 1). Previous studies [54,55] intriguingly suggest that the Maillard reaction products, a result of heat treatment, significantly enhance the antioxidant values of lipophilic molecules compared to untreated flour. This trend, however, needs to be evident in the other two series, underscoring the unique properties of acorn “coffee” and “super coffee”.

Interestingly, the sums of the total phenol content (TPC) and total reduction potential (TRP) values for all three applied solvents gravitate toward the same values regardless of the type of sample tested. Thus, the sums of TPC for acorn flour, “coffee”, and “super coffee” were 89.2, 92.9, and 87 mg GAE/g dw, respectively, while the sums of TRP are 6.86, 7.0, and 6.99 mg AAE/g dw, respectively (Figure 1a and Figure 3b).

Thermal treatment raised the TPC in methanol extracts by 15% (57.8 5 mg GAE/g dw to 66.5 mg GAE/g dw) and in acetone by 9%, while flavonoids decreased (methanol −17%, acetone −4%), mirroring findings that heat degrades tannins but increases non-tannin phenolics like gallic acid in *Q. robur* acorn products.

The findings suggest that the antioxidant activity of the studied samples is in correlation with the total content of phenols and flavonoids. This is a consequence of the fact that methanol extracts showed the highest values of antioxidant activity (~127 for ABTS, ~584 CUPRAC, ~200 for FRAP, and ~5.0 for TRP in suitable units), while the hexane extracts showed the lowest values (Table 1). Antioxidant assays (ABTS, DPPH, CUPRAC, FRAP, TRP) showed activity that correlated strongly with phenolic and flavonoid levels (Table 1). Methanol extracts were of the highest antioxidant capacity (e.g., ABTS ~127 mg TE/g dw), while hexane extracts were lowest, paralleling trends in *Q. robur* oil studies where hydrophilic phenolics dominate antioxidant activity [51,56]. Unexpectedly, the DPPH assay showed higher values for acetone extract (104 mg TE/g dw) than methanol extract (81 mg TE/g dw) (Figure 2b) [56], which is comparable with the findings of Kim et al., for *Q. acuta* Thunb extracts [57]. Finally, FRAP indicated increased reducing capacity post-roasting, especially in less polar solvents (methanol +100%, acetone +124%, hexane +375%), supporting evidence that Maillard reaction products and lipophilic antioxidants enhance reducing power after heat processing [7].

Heat triggers Maillard reactions between reducing sugars and amino acids, resulting in the formation of high-molecular-weight melanoidins. These compounds contribute strong antioxidant properties through electron donation and radical scavenging [5,58,59]. Despite the degradation of some native phenolics, the formation of melanoidins slightly increases the antioxidant capacity of hexane extracts (DPPH, ABTS, and FRAP assays), which better solubilize these complex compounds. Roasting partially degrades chlorogenic and complex phenolics into simpler, more bioavailable antioxidants (e.g., gallic and caffeic acids), enhancing results by assays like ABTS and FRAP. Polar solvents retain more of these smaller phenolics, increasing measured antioxidant activity following heat treatment [59].

All correlations between antioxidant activity assays and total phenolic and total flavonoid content are statistically significant at *p* < 0.05. Comparing correlations between total phenolic content and antioxidant activity assays, it can be concluded that the strongest correlation was observed between the CUPRAC and TPC assays (r = 0.99). Regarding the total flavonoid content, the strongest correlation was also observed between CUPRAC and TFC (r = 0.98). Significant correlations between antioxidant activity assays and total phenolic and flavonoid content strongly imply that phenolic compounds have the highest impact on the overall antioxidant activity of all analyzed samples.

**Figure 4:**
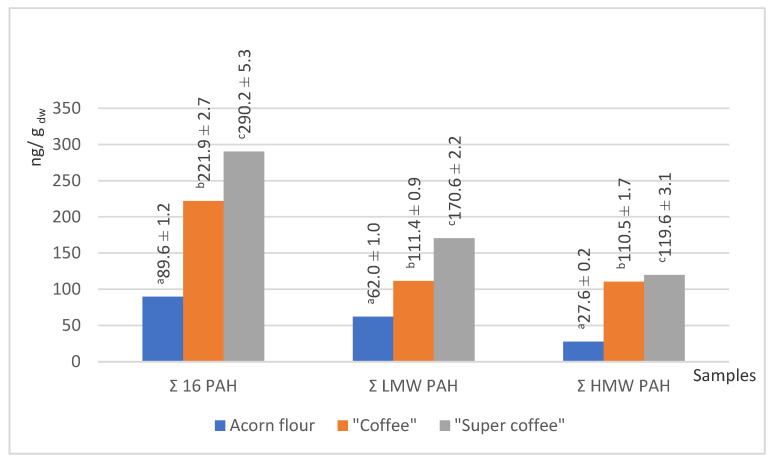
The total content of 16 priority PAHs, LMW, and HMW PAHs in acorn samples. Groups marked with different letters (a, b, c) are significantly different at *p* < 0.05.

**Figure 5:**
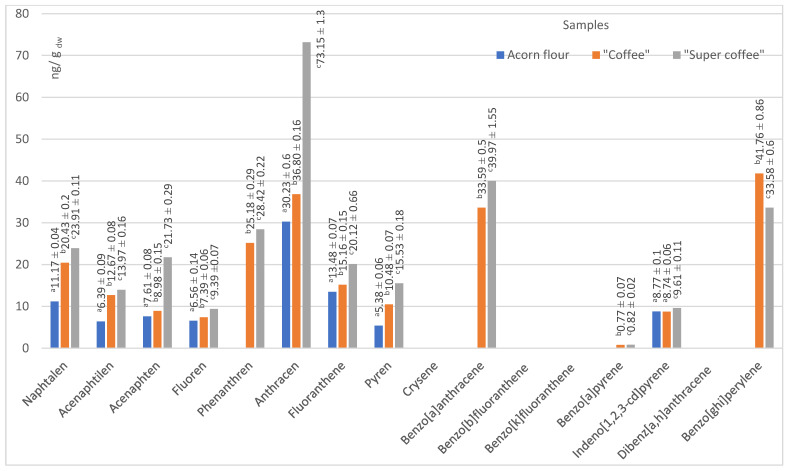
Content of 16 priority PAHs by species, in native acorn flour, “coffee”, and “super coffee” samples. Groups marked with different letters (a, b, c) are significantly different at *p* < 0.05.

The process of coffee roasting leads not only to a significant change in the content of dry matter but it also enhances the formation of volatile pyrolysis products, which include potentially harmful PAHs. This process may also cause the degradation of polysaccharides and amino and chlorogenic acids [7]. Therefore, the results concerning the amount of PAHs in our samples highlight some of the health implications of acorn use in the human diet as a natural substitute for coffee. The following content of PAHs in the tested acorn samples was observed (Figure 4 and Figure 5).

Two of our acorn samples (“coffee” and “super coffee”) that were exposed to high temperature have been subject to the phenomenon that thermal treatment may significantly increase the content of PAHs. The process of exposing raw acorns to temperatures higher than 200 °C (“roasting”) may have caused the pyrolytic breakdown of lipids, polysaccharides, and proteins, thereby enabling the generation of PAHs [60]. In our case, “coffee” and “super coffee” samples had more PAHs by 2–3 times compared to the native acorn powder (flour). Such a phenomenon is particularly evident in the “super coffee” sample, since both low molecular weight (LMW) and high molecular weight (HMW) PAHs were present. The presence of PAHs in native acorn powder (“flour”) has not been hypothesized since the sample was not exposed to high heat. Therefore, we suggest the present results are due to the contamination at the source. We are prone to further understanding of this increase in the level of PAHs as a crucial argument for potential health risks, since high levels of PAHs may have negative health implications. The contents of all PAH species in our samples are presented in Figure 5.

Data from the literature assured us that values that we have obtained for total 16 PAH for our two investigated acorn samples, “coffee” (221.9 ng/g dw) and “super coffee” (290.2 ng/g dw), are in accordance with previous findings of Sadowska-Rociek et al. [61] for coffee substitute, natural instant, and natural roasted coffee (200.70–308.80; 215.90–464.40 and 224.70–459.00 ng/g dw, respectively). These values are significant as they indicate the range of PAHs in these products, which may impact food safety and consumer health. Another study on the PAH content in coffee samples [62] revealed that chrysene and pyrene were the predominant PAHs in coffee, reaching up to 95.6 ng/g and 404.7 ng/g, respectively. Benzo[a]pyrene appeared in dark roasts in a concentration of 9.0 ng/g.

Anthracene was the dominant PAH across all samples. In “coffee” and “super coffee,” benzo[a]anthracene, benzo[g,h,i]perylene, and phenanthrene contributed substantially to total PAHs. None of these compounds were detected in the native flour, implying that roasting was the source. However, the detection of indeno[1,2,3-cd]pyrene in flour (8.8 ng/g dw) suggests some environmental contamination [31].

Approximately 30% of PAHs are typically transferred to the infusion; the rest remains in the ground [62].

The presence or absence of these specific PAH species is significant, as they are known to have different health effects. Therefore, understanding the distribution of these PAH species may enlighten risk assessments and safety measures.

“Acceptance marker” PAH4 (sum of four different polycyclic aromatic hydrocarbons, named benzo[a]anthracene, chrysene, benzo[b]fluoranthene, and benzo[a]pyrene) in these samples depends primarily on the content of benzo[a]anthracene because the content of benzo[a]pyrene is relatively low. In contrast, the other two compounds, chrysene and benzo[b]fluoranthene, were not detected in any of the samples. Thus, raw acorn flour does not contain any of the mentioned compromised hydrocarbons from the PAH4 group, while “coffee” and “super coffee” contain 34.3 ± 0.6 and 39.8 ± 0.6 ng/g, respectively.

On the other hand, in the study mentioned above, ref. [61] confirmed the content of PAH4 compounds in almost all samples of coffee and substitutes, while Orecchio et al. [63] also determined the presence of all PAH4 in the tested samples of coffee from the supermarket, finding phenanthrene and its methyl derivatives as the most abundant.

Of the PAHs in raw acorn flour, only indeno[1,2,3-cd]pyrene was detected in a concentration of 8.8 ± 0.1 ng/g dw.

## 4. Conclusions

Our study offers valuable insights into the benefits and potential risks associated with incorporating acorns into the human diet. We found that both native and heat-treated acorn powders contain significant amounts of total polyphenolic compounds, with the highest levels extracted by highly polar solvents. Encouragingly, heat-treated samples (“coffee” and “super coffee”) actually showed an increased polyphenol content, which suggests enhanced potential health benefits. However, it is important to note that thermal processing decreased the total flavonoid content, especially since the highest flavonoid levels were detected in methanol extracts and the lowest in hexane extracts.

When it comes to antioxidant activity, methanol extracts consistently outperformed acetone and hexane extracts, except in the DPPH assay, where acetone extracts showed higher activity—a detail that warrants further clarification.

On the safety side, roasting significantly raised the levels of polycyclic aromatic hydrocarbons (PAHs), with the “coffee” and “super coffee” samples showing two to three times more PAHs than the raw acorn powder (“flour”). Despite this increase, the carcinogenic PAH4 marker was absent in the native flour, indicating that unprocessed acorn powder is safe for dietary use. Additionally, the levels of specific PAHs, including benzo[a]pyrene, in the roasted samples remained well below the EU Commission’s limits set for cocoa products, indicating a low overall health risk. The only possible concern might be related to anthracene content.

Because the EU has not established PAH standards for regular coffee, a direct comparison between our acorn-based products and traditional coffee is not straightforward. However, our findings suggest that PAH levels in acorn “coffee” and “super coffee” are within acceptable limits and notably lower than many coffee and coffee substitute products available on the market.

In summary, native acorn powder is a rich source of antioxidants that can effectively neutralize harmful free radicals. Roasting induces a complex interplay of effects, like the thermal breakdown of some phenolics and the formation of Maillard reaction products with antioxidant properties, alongside the formation of PAHs. This balance, combined with solvent extraction characteristics, helps explain the antioxidant behavior and PAH levels we observed.

Given the low levels of PAHs even after roasting, acorns show promise as a safe alternative to traditional coffee. Our results highlight that the overall benefit-to-risk ratio favors the use of acorns in the diet, either raw or roasted. While thermal processing enhances the antioxidant activity of acorn-based products, it also introduces measurable amounts of PAHs. However, these remain within acceptable regulatory limits for similar food categories. Thus, *Q. robur* acorns, particularly in their native or moderately roasted form, represent a promising functional food ingredient with minimal safety concerns when properly processed.

## Figures and Tables

**Table 1:** Correlation coefficients between antioxidant assays, TPC, and TFC.

	CUPRAC	DPPH	TFC	FRAP	TPC	TRP
ABTS	0.90 *	0.94 *	0.88 *	0.95 *	0.88 *	0.90 *
CUPRAC		0.85 *	0.98 *	0.90 *	0.99 *	1.00 *
DPPH			0.82 *	0.85 *	0.82 *	0.84 *
TFC				0.87 *	0.97 *	0.98 *
FRAP					0.88 *	0.90 *
TPC						0.99 *

* Marked correlations are significant at *p* < 0.05.

## Data Availability

The original contributions presented in this study are included in the article. Further inquiries can be directed to the corresponding authors.

## Abbreviations

The following abbreviations are used in this manuscript:

TPC	Total phenolic content
TFC	Total flavonoid content
PAHs	Polycyclic aromatic hydrocarbons
GAE	Gallic Acid Equivalent
LMW, MMW and HMW	Low-, medium-, and high-molecular-weight
EPA	Environmental Protection Agency
Nap	Naphthalene
Acy	Acenaphthylene
Ace	Acenaphthene
Flr	Fluorene
Phe	Phenanthrene
Ant	Antanthracene
Flt	Fluoranthene
Pyr	Pyrene
BaA	Benzo[a]anthracene
CHR	Chrysene
BbF	Benzo[b]fluoranthene
BkF	Benzo[k]fluoranthene
BaP	Benzo[a]pyrene
DhA	Dibenz[a,h]anthracene
BgP	Benzo[g,h, i]perylene
IcP	Indeno[1,2,3-cd]pyrene
IARC	International Agency for Research on Cancer
PAH4	Sum of four polycyclic aromatic hydrocarbons, named: benzo[a]anthracene, chrysene, benzo[b]fluoranthene, and benzo[a]pyrene)
MAQs	Maximum Allowable Quantities
SIM	Selected ion monitoring
DPPH	2,2-Diphenyl-1-picrylhydrazyl
ABTS	2,2′-Azino-bis(3-ethylbenzothiazoline-6-sulphonic acid)
DMSO	Dimethyl sulphoxide
RE	Rutin equivalents
TE	Trolox equivalents
CUPRAC	Cupric reducing antioxidant capacity
FRAP	Ferric-reducing antioxidant power
TPZT	2,4,6-tris(2-pyridyl)-s-triazine
TRP	Total reducing power
AAE	Ascorbic acid equivalents
QuEChERS	Quick, Effective, Cheap, Easy, Rugged, Safe
GC–MS	Gas chromatography–mass spectrometry
LOD	Limit of detection
LOQ	Limit of quantitation
HP-5MS	5% Phenyl Methyl Siloxane
RP-HPLC-ESI-TOF	Chromatography-electrospray ionisation time-of-flight
